# Consultants and Press Announcements

**Published:** 1914-03-21

**Authors:** M.D. Lond

**Affiliations:** London


*To the Editor of* The Hospital.

### Consultants and Press Announcements.

*To the Editor of* The Hospital.

Sir,—Within the last three weeks those members of the medical profession who have at heart the interests of their calling have been inexpressibly shocked at seeing in the newspapers an advertisement of a work on domestic medicine in which the names of their leaders appeared in very bold type and further distinguished by an array of academic letters. The vulgarity of this announcement was such that it even penetrated that apathy which seems to be a characteristic of the time, with the result that several protests have been made in the medical press. Moreover, as a result of these protests, we are told that no fresh advertisements of the same kind will be ordered and that no future announcements mentioning the names of the distinguished baronets, knights, physicians, surgeons, and specialists—the exact number of whom I forget for the moment and really have no wish to remember—shall appear. But, remarkably enough, we have also been told in the most casual manner that it was too late to stop the appearance of some advertisements of the same objectionable kind which had been already arranged, which apparently means that for a longer or shorter period, dependent entirely on the original advertisement campaign of the publishers, the public may possibly be further gratified by seeing in prominent type the distinguishing qualifications of a number of consultants in whom it may or may not be interested.

Surely the matter is not to be allowed to rest at this point. Surely the initial blatancy of the announcement in question is only equalled by the pitiable feebleness of the protest so far made against it. As an observant and interested member of the profession it is to me an amazing thing that one, some, or all of those baronets, knights, physicians, surgeons, and specialists should not have moved heaven and earth to prevent the further appearance of a notice which, as has been admitted by the editor of the publication mentioned, directly contravened the ethics of the medical profession. Up to the present time the average layman who has had time to think about these things has always considered that the leaders of the medical profession do not advertise in the usually accepted sense of that term. In recent years he has been pleased to see medical subjects more freely discussed than before in the lay press, he has liked to read sane and well-thought-out interviews between responsible medical men and the representatives of lay journals in regard to questions of public health and interest; but it is quite a mistake to suppose that the educated Englishman has ever been seriously impressed by blatant interviews or vulgar indirect advertisements of medical men. It is quite a mistake to suppose that he has been led to pour his guineas into the coffers of any self-constituted specialist at the sound of a press trumpet. He has always been confident that the best men only permit their names to appear in a certain reputable way. Unfortunately, if it is now observed that one of the most lamentable advertisements that has ever profaned the dignity of the medical profession in this country is allowed to pass with but the feeblest measure of protest, and without apparently the slightest active resistance, the observant citizen must inevitably conclude that the members of that body have decided to depart from the time-honoured paths of reticence and honour, to come down into the market-places and shout their wares amongst the corn-chandlers, pill-makers, and soap vendors. Clearly, he will argue, the doctors have a perfect right to descend to the open market if they like, but that when they do so they must resign all claim to be practitioners of an art, and must expect to be ranked as men of trade. Trade is honourable indeed, and may be pursued by men of honour, but the most ardent defender of commerce gives it second place to art, and it has always been one of the boasts of the disciples of Æsculapius that they follow the noblest art of all.

The question then is, "Are we for the future to be artists or tradesmen in the eyes of the world?" And, to use a colloquialism, it is up to those distinguished baronets, knights, physicians, surgeons, and specialists whose names have so lately adorned the advertisement columns of the lay press to make it clear to the public that in spite of appearances they stand for art and not for trade. They can only do so by making a spirited protest at every public reference to any work of domestic medicine in which their names appear, or in which their work is boomed directly or indirectly; they can only carry their point by protesting with equal warmth against the booming of any work in which their names, researches, or distinctions have at any time been used as a trade advertisement.

It is quite ineffectual to confine protests to a few short letters in technical journals. To put the matter right in the eyes of the educated layman it is necessary that some more public action be taken to show the position of the profession in the matter. Here is an opportunity for the Royal Colleges of Physicians and Surgeons to come to the aid of their fellows and members whose honour has been impugned, and to use their influence and wealth to put this matter right. To any active medical man it is grievous to see the dignitaries of the Royal Colleges, enthroned in majesty and apparent might, issuing decrees with the magnificence of Cæsars and in the language of Jove, whilst such decrees are concerned with the question as to whether Jones shall style himself "Dr." or "Mr.," or as to the amount of Latin Smith shall cram up before being admitted to their august membership; whilst the Olympians fail to bother themselves with the great living issues which concern the rank and file of the profession, as in the earning of their daily bread, and more, in the protection of their honour as professional men. Here is an opportunity for the Colleges and Councils to wake up and support by active measures the protests of the distinguished gentlemen whose names have been scattered broadcast throughout the land in this unfortunate manner.—Faithfully yours, M.D.Lond.

London, March 16, 1914.

